# Biochemical Recurrence in Prostate Cancer Is Associated with the Composition of *Lactobacillus*: Microbiome Analysis of Prostatic Tissue

**DOI:** 10.3390/ijms241310423

**Published:** 2023-06-21

**Authors:** Jae Heon Kim, Hoonhee Seo, Sukyung Kim, Asad Ul-Haq, Md Abdur Rahim, Sujin Jo, Ho-Yeon Song, Yun Seob Song

**Affiliations:** 1 Department of Urology, School of Medicine, Soonchunhyang University, Seoul 04401, Republic of Korea; 2 Department of Microbiology and Immunology, School of Medicine, Soonchunhyang University, Chungnam 31151, Republic of Korea; 3 Probiotics Microbiome Convergence Center, Soonchunhyang University, Chungnam 31538, Republic of Korea

**Keywords:** prostate carcer, cancer stem cells, tumor microenvironment, biomarker, therapeutic targets, drug resistance, cancer prevention

## Abstract

Many human pathologies, such as malignancy, are linked with specific bacteria and changes in the constituents of the microbiome. In order to examine the association between an imbalance of bacteria and prostate carcinoma, a comparison of the microbiomes present in patients with biochemical recurrence (BCR) or NO BCR (NBCR) was performed. Additionally, 16S rRNA-based next-generation sequencing was applied to identify the bacterial profiles within these tumors in terms of the bacteria and operational genes present. The percentage average taxonomic composition between the taxa indicated no difference between BCR and NBCR. In addition, alpha and beta diversity indices presented no distinction between the cohorts in any statistical method. However, taxonomic biomarker discovery indicated a relatively higher population of *Lactobacillus* in the NBCR group, and this finding was supported by PCR data. Along with that, differences in the operational activity of the bacterial genes were also determined. It is proposed that the biochemical recurrence was linked to the quantity of *Lactobacillus* present. The aim of this study was to investigate the microbiome involved in prostate carcinoma and the potential association between them.

## 1. Introduction

Humans have a mutually beneficial association with over 100 trillion microbial cells, which reside within their bodies [1]. It is well established that bacteria in specific body parts influence pathogenesis, immune system performance, and long-term health [2]. The development of high-throughput next-generation sequencing (NGS) methods has increased the amount of research on the function of the human microbiome concerning numerous illnesses or disorders [1,3].

Prostate cancer ranks among the most frequently occurring cancers in males [4,5]. Prostate biology may be influenced by viral and bacterial infections, inflammatory triggers and environmental factors like nutrition and lifestyle [6,7,8,9]. The members of the local microbiota community, their interactions, and how they interact with the human host impact human physiological processes and general well-being [10,11,12]. It is conceivable that these bacteria may generate or contribute to an inflammatory process within cancerous prostate tissue, although the exact mechanisms involved are not fully understood. Changes in the composition of bacterial communities have been observed in prostate cancer, suggesting their potential role in promoting proinflammatory responses or modifying the environment within the gland, thus influencing the development of cancer [13,14,15].

Opportunistic bacteria are thought to promote the inflammatory process within the prostate. Among them, endogenous Enterobacteriaceae, such as *Escherichia coli* or *Pseudomonas* spp., and sexually transmitted bacteria, such as *Neisseria gonorrhoeae*, *Chlamydia trachomatis*, and *Trichomonas vaginalis*, are considered to be the most to blame [16,17]. Heightened visceral inflammation has mainly been demonstrated in prostate carcinoma specimens in the presence of *Propionibacterium acnes* [14,17]. Despite these reports, a complete and in-depth description of the microbiome within diseased and normal prostate samples has yet to be published [10,18,19]. Previous studies have examined the microbiome of the male genital tract, including the analysis of prokaryotic and viral DNA sequences in prostate carcinoma samples. However, these reports are now considered inadequate and lacking in comprehensive and informative data, given the recent advancements in high-throughput sequencing and bioinformatics technologies [20,21].

Many human pathologies, such as malignancy, are linked with specific bacteria and changes in the constituents of the microbiome. In order to examine the association between an imbalance of bacteria and prostate carcinoma, a comparison of the microbiomes presents in patients with biochemical recurrence (BCR) or NO BCR (NBCR), respectively, was performed. The current research included an assessment of the specimens from BCR, which were linked with a different microbiome to those from NBCR. The NGS technique was utilized to assess the relevance of the underlying disease processes connected to prostate cancer.

Here, we determined the potential role of microbiome affecting prostate carcinoma and studied the association between them using 16S rRNA next-generation sequencing technology. We expect that the findings of this study will be a valuable addition to the already available literature on microbiome and prostate cancer research.

## 2. Results

### 2.1. Patient Profiles

The study population included 26 patients with prostate carcinoma, of which 13 had BCR and 13 had NBCR (Tables S1 and S2). Figure S1 illustrates Kaplan–Meier curves for survival.

### 2.2. Sample Analysis and Integrity of the Sequenced Data

The Illumina system was utilized for the sequencing of 26 specimens. The poor-quality data created by the NGS sequencing platform was discarded using a prefilter system. Finally, the valid reads were included in the data following the elimination of amplicons that were non-specific or not allocated to designated taxa or chimeras. After that, distinct illustrative sequences were categorized into phylum, classes, orders, families, and genera levels. The 16S rRNA sequences identified in this study appeared to reflect most of the bacterial sequences present in the specimens, according to the value of Good’s estimator of coverage (Table S2).

### 2.3. Bacterial Taxa (BCR vs. NBCR)

**Figure 1:**
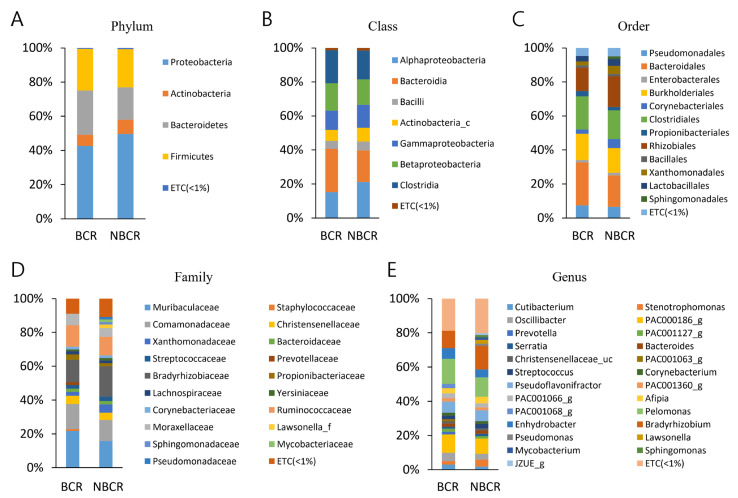
Averaged taxonomic composition for tumor regions in the BCR and NBCR group. Taxonomic relative abundance was classified at the (**A**) phylum, (**B**) class, (**C**) order, (**D**) family, and (**E**) genus level, and relative abundances of less than 1% were expressed as ETC. Wilcoxon rank-sum test was used to analyze the significance between the two groups. There were no significant differences.

The populations of bacteria from the BCR and NBCR samples underwent analysis concerning the taxonomic hierarchy (Table S2, Figure 1). The leading three phyla in abundance, which made up 93.0% of sequences in the BCR specimens, were Proteobacteria, Bacteroidetes, and Firmicutes. The same bacteria formed 91.0% of sequences within the NBCR samples. The most frequently arising phylum was Proteobacteria, which occurred in BCR and NBCR sequences with a frequency of 42.6% and 49.7%, respectively. No significant differences between the phyla present within the BCR and NBCR samples were identified (Table S2, Figure 1).

There were no differences in the identified classes between BCR and NBCR cohorts (Table S2, Figure 1); the leading five classes of bacteria were *Alphaproteobacteria*, *Bacteroidia*, *Clostridia*, *Betaproteobacteria*, and *Gammaproteobacteria*, making up 87.4% and 85% of sequences, respectively. The most frequently observed classes were *Bacterioidia* (25.4%, BCR) and *Alphaproteobacteria* (21.2%, NBCR). The order distribution in both BCR and NBCR specimens was similar (Table S2, Figure 1). *Bacteroidales*, *Clostridiales*, *Burkholderiales,* and *Rhizobiales* were the most common orders seen in both BCR and NBCR, forming 74.0% and 68.0% of the sequences, respectively. *Bacteriodales* was the most commonly observed, with a frequency of 25.4% and 18.5% in BCR and NBCR, respectively.

A total of 62.6% and 57.0% of all sequences in BCR and NBCR, respectively, encompassed the families, *Muribaculaceae*, *Comamonadacea*, *Bradyrhizobiaceae,* and *Ruminococcaceae*, with the former being the most frequently (21.7%) noted family in BCR, and *Bradyrhizobiaceae* the most commonly (17.7%) seen in NBCR. The distribution of families was equivalent between the two sample cohorts (Table S2, Figure 1).

The three most frequently arising genera within BCR (35.6% sequences) were *Pelomonas*, *PAC000186_g,* and *Bradyrhizobium*. In the NBCR samples, 25.1% of the sequences comprised two genera, *Bradyrhizobium* and *Pelomonas*. The most common genera in the BCR and NBCR cohorts were *Pelomonas* (14.5%) and *Bradyrizobium* (13.8%), respectively. The genera distribution between the two specimen groups was similar (Table S2, Figure 1).

### 2.4. Richness and Diversity (BCR vs. NBCR)

**Figure 2:**
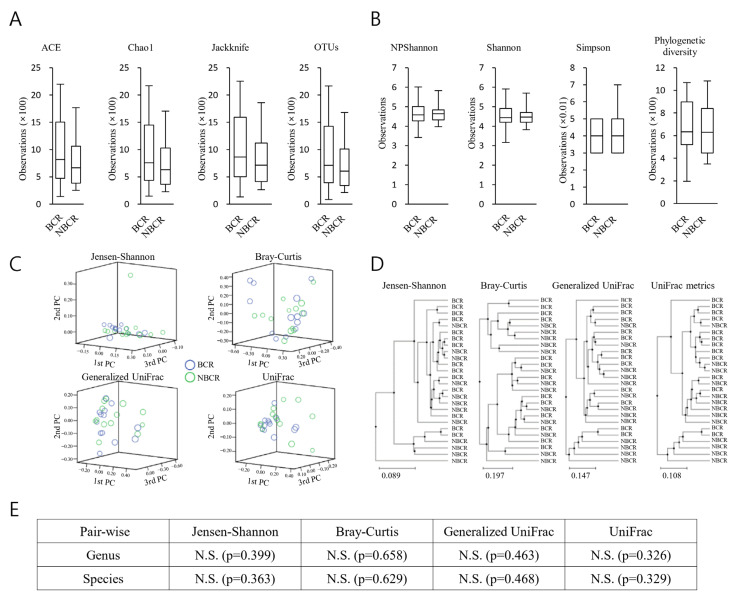
Alpha and beta diversity indices for BCR or NBCR. (**A**) Species richness was analyzed with Ace, Chao1, Jackknife, and OTUs. (**B**) The species diversity was examined using NPShannon, Shannon, Simpson, and Phylogenetic diversity. The boxplot edges denote the first and third quartiles, while the thick black band in the horizontal direction depicts the median value. The alpha diversity study results did not reveal any significant differences. (**C**) Distances between communities were analyzed by a principal coordinate analysis (PCoA). (**D**) The Unweighted Pair Group Method with Arithmetic Mean (UPGMA) was used to analyze clustering. (**E**) Beta set significance was demonstrated by Permutational multivariate analysis of variance (PERMANOVA). The Jensen–Shannon, Bray–Curtis, Generalized UniFrac, and UniFrac metrics were used to analyze beta diversity. N.S., not significant.

There were trends for the BCR specimens to exhibit a greater bacterial richness than those from NBCR (Figure 2A). In contrast, the NBCR specimens showed greater diversity than the BCR samples (Figure 2B). However, these observations failed to reach significance (Figure 2C).

Permutational multivariate analysis of variance (PERMANOVA) was employed in order to calculate beta set significance (Figure 2D). Jensen–Shannon, Bray–Curtis, Generalized UniFrac, and UniFrac metric-based beta diversity analyses each failed to identify any significant dissimilarities (Figure 2E).

### 2.5. Taxonomical Biomarker (BCR vs. NBCR)

**Figure 3:**
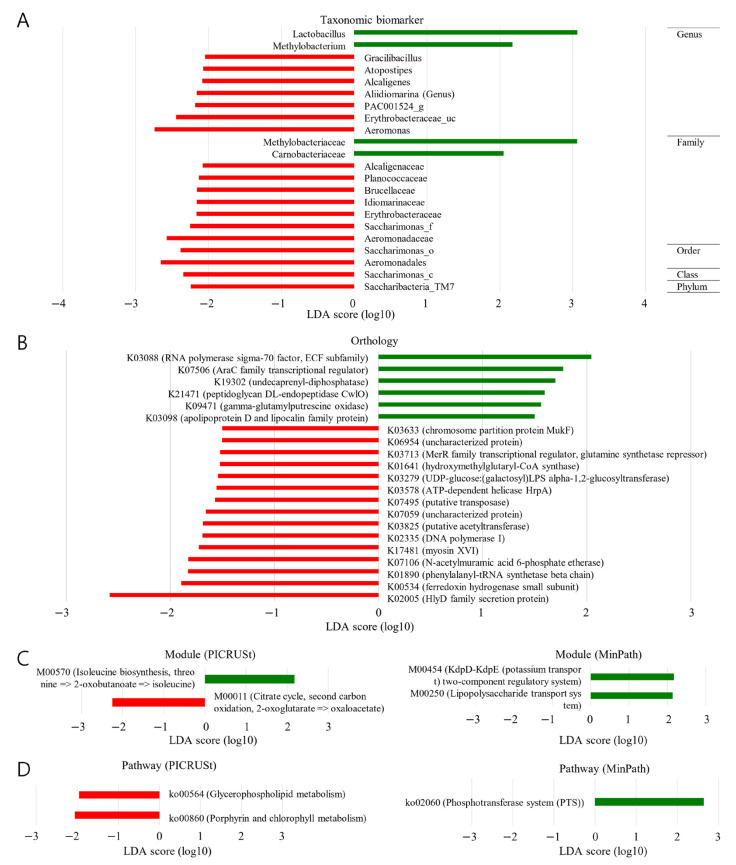
Discovery of taxonomic and functional biomarkers for BCR or NBCR using LEfSe (Linear discriminant analysis Effect Size). (**A**) Taxonomic and (**B**–**D**) functional biomarkers were analyzed in ortholog, module, and pathway, respectively. PICRUSt (Phylogenetic Investigation of Communities by Reconstruction of Unobserved States) and MinPath (Minimal set of Pathways) techniques were used to determine the module and pathway. The KEGG (Kyoto Encyclopedia of Genes and Genomes) database was employed for functional biomarker analysis. The red area indicates more abundance in the BCR group, and the green area is vice versa.

Different bacterial constituents were noted within the two groups of samples (Figure 3A). The BCR specimens were observed to be enriched within the following taxonomic strata as follows: phylum, *Sacharibacteria_TM7* (LDA score ≤ −2); class, *Sacharimonas_c* (LDA score ≤ −2); order, *Sacharimonas_o* and *Aeromonadales* (LDA score ≤ −2); family, *Alcaligenacea*, *Planococcaceae*, *Brucellaceae*, *Idiomarinaseae*, *Erythrobacteracea*, *Sacharimonas_f* and *Aeromonadaceae* (LDA score ≤ −2). In contrast, enrichment at the family stratum in the NBCR specimens was seen concerning *Methylobacteracea* (LDA score ≥ 2) and *Carnobacteracea* (LDA score ≥ 3).

### 2.6. Functional Biomarker (BCR vs. NBCR)

Malignant lesions were notably populous in those associated with genetic information processing. The functional characteristics of the microbiome components linked with prostatic malignancy were analyzed using PICRUSt (Figure 3B–D). The glycophospholipid, porphyrin, and chlorophyll metabolism pathways occurred with a greater frequency in the BCR samples (LDA score ≤ −2).

### 2.7. Quantitative Evaluation of Lactobacillus Abundance (BCR vs. NBCR)

**Figure 4:**
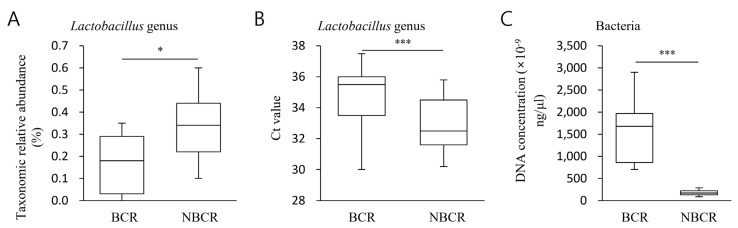
Taxonomic abundance for *Lactobacillus* with the largest LDA effect size between NBCR or BCR. (**A**) Taxonomic relative abundance showed *Lactobacillus* increased. (**B**) The quantitative distribution of *Lactobacillus* was also confirmed through quantitative real-time PCR analysis. The Ct value in the NBCR group sample was lower than that of the BCR group, thereby confirming that *Lactobacillus* was contained more in the NBCR than in the BCR. (**C**) PCR also quantified the number of total bacteria, and it was confirmed that the BCR contained significantly higher levels than the NBCR group. Statistical significance with controls was analyzed using Wilcoxon rank-sum test and unpaired Student’s *t*-test. * *p* < 0.05, *** *p* < 0.001.

An analysis of the taxonomic quantity of *Lactobacillus* with a notable LDA effect size was performed, comparing NBCR and BCR specimens. Relative median values for NBCR and BCR were 0.34% and 0.18%, respectively (*p* < 0.05), indicating a larger relative *Lactobacillus* population in the former (Figure 4A). The corresponding absolute median values were 35.2 and 32.6, respectively (*p* < 0.01, Ct value), indicating a higher quantitative taxonomic *Lactobacillus* population in NBCR specimens (Figure 4B). The equivalent mean values for quantitative assessments of total bacteria were 1544.2 and 58.0, respectively (*p* < 0.01) (Figure 4C).

## 3. Discussion

The potential link between microbes and the various tumorigenesis stages has received much interest since the World Health Organization classified *Helicobacter pylori* as a carcinogen [22]. Scant data define these microorganisms’ role in the disease pathways driving prostate neoplasia, even though 15.4% of human tumors have been related to disease-inducing pathogens [23]

A regional microbiome particular to the prostate gland has been described [24]. Microorganisms are abundant within the prostate; this observation may imply a potential association between the local bacterial population’s components and the malignancy per LEfSe [25]. In the current study, the regional microbiota, specific to the prostate, could be an additional prognostic factor.

In the current work, the most frequently arising findings within the taxonomic hierarchy were as follows: phyla, *Proteobacteria* in both BCR and NBCR; class, *Bacteroidia* and *Alphaproteobacteria* in BCR and NBCR, respectively; order, *Bacteroidales* in BCR and NBCR; family, *Muribaculaceae* and *Bradyrhizobiaceae* in BCR and NBCR, respectively; genus, *Pelomonas* and *Bradyrhizobium* in BCR and NBCR, respectively. No significant differences were observed between the groups. Thus, regional prostate-specific microbiota was identified, but these were similar in composition within both BCR and NBCR.

For each taxonomic stratum, a rising trend in the bacterial population of the specimens from BCR patients, as opposed to those from NBCR patients, could be seen. Additionally, the heterogeneity of the bacteria appeared to increase within the BCR specimens but not in the NBCR specimens. However, neither of these observations reached statistical significance.

No dissimilarities between the bacterial populations within BCR and NBCR samples were observed following PCA concerning the OTU strata. The clustering results (UPGMA) indicated no individual clustering between the BCR and NBCR specimens, implying that the general configurations of the bacterial populations within the groups were similar. Additionally, after beta diversity analysis, no differences were noted between BCR and NBCR samples. In the current work, biochemical recurrence was not linked to the heterogeneity of the microenvironment of the microbiome.

The results from the LEfSE analysis revealed that the NBCR specimens were enriched at the family stratum concerning *Carnobacteracea* (LDA score ≥ 2). The genera, *Gracilibacillus*, *Atopostipes*, *Alcaligenes*, *Aliidiomarina*, *PAC001524_g*, *Erythrobacteriaceae_uc,* and *Aeromonas,* demonstrated enrichment in the BCR specimens (LDA score ≥ 3). Thus, based on the study’s findings, we can assume that biochemical recurrence might be associated with various microbiome compositions.

The practical consequences of the presence of *Lactobacillus* in various pathologies are well-studied [26,27]; pre-clinical work has demonstrated the capacity of this bacterium to diminish the chronic inflammatory process that accompanies carcinogenesis [28,29]. In individuals with colorectal carcinoma, the amount of *Lactobacillus* was notably diminished in fecal specimens [30], implying that it may participate in malignant suppression. The gastrointestinal microbiota is altered by *Lactobacillus gallinarum*, a bacterium synthesizing anti-tumor factors to offer prophylaxis against the onset of colorectal neoplasia [31].

In the current work, we selected *Lactobacillus* for detailed study because *Lactobacillus* was identified as the most prominent functional biomarker (LDA scores ≥ 3). Additionally, our PCR results also comply with these findings. Furthermore, the biochemical recurrence was related to the size of the *Lactobacillus* population. The microbiome within the prostate is influenced by the presence of *Lactobacillus,* which manufactures anti-tumor factors to confer protection concerning the advancement of prostate carcinoma. This study demonstrated a greater population in BCR samples from a quantitative perspective. The quantity of *Lactobacillus* was probably linked with biochemical recurrence. Additionally, a significantly higher bacterial population was observed in BCR that complies with previous studies [32,33].

Several studies reported that *Lactobacillus* has a positive impact in lowering the risk of postoperative complications among individuals with cancer [34,35]. Another study showed the potential of *Lactobacillus* to decrease the carcinogenic biomarkers in colorectal cancer animal models [36]. These findings collectively suggest the significance of *Lactobacillus* in the context of prostate cancer, indicating a possible connection between the population of *Lactobacillus* and the occurrence of biochemical recurrence in patients.

Genus *Methylobacterium* was identified as a second functional biomarker (LDA scores ≤ 3) in NBCR than BCR which is aligned with some previous studies [37,38]. On the contrary, some researchers have indicated that the species *Methylobacterium* radio-tolerant is relatively enriched in tumor tissue [39,40]. So, detailed research is needed to evaluate the exact role of *Methylobacterium* in cancer.

An increased abundance of pathways associated with glycophospholipid, porphyrin, and chlorophyll metabolism was shown in the BCR samples following PICRUSt analysis. The grade of the malignant lesion was linked to a different functional microbiome within this work.

Our study has certain limitations; firstly, we could only manage a small number of subjects for this study. Secondly, the specimens were collected from a geographically confined area. Thirdly, we could not analyze specimens according to different ages or race groups. Moreover, the role of *Lactobacillus* in controlling prostate carcinoma needs to be explored using the humanized mouse model. Despite the limitations, we expect that our study will be a valuable addition to understanding prostate carcinoma in the prospect of microbiomes. Until now, it has been accepted that there is no symbiotic microbiome in prostate tissue, especially in healthy prostate tissue. In this case, where does the microbiome reported in the diseased prostate come from? In order to answer this question, we will need microbiome studies using both urine and tissues in the future.

To summarize, bacterial content was changed within the specimens acquired from BCR, with compositional and gene functional differences observed when contrasted against specimens taken from patients with NBCR. It was noted that *Lactobacillus* was the most frequently present genus within NBCR.

## 4. Materials and Methods

### 4.1. Subject Recruitment and Sample Collection

Prostate cancer patients (*n* = 26) from the Urology Department were enrolled in the study (Table S1). This is the same as a part of the patient subjects in the study already conducted by this research team [41]. The patients selected for this study underwent radical prostatectomy followed up for more than 5 years from prostate cancer and paraffin tissues were obtained through surgery. The cancer and non-cancer areas around prostate cancer were micro-dissected and then NGS was performed. Prostate cancer patients whose 16S rRNA measurements passed the QC were eligible for microbiome analysis. The prostate cancer samples were obtained from individuals who did not have any significant coexisting conditions such as diabetes mellitus, immunodeficiency, or genetic disorders. These individuals had not received any treatment for their prostate tumor, and they had refrained from taking antimicrobial medications for at least 14 days prior to the collection of the specimens. The median age of the study population was 72.5. The research was carried out in accordance with the principles outlined in the Declaration of Helsinki [42], and received approval from the Ethics Committee of Soonchunhyang University, Korea (approval number: 2017-02-002). According to the biochemical recurrence, prostate cancer patients were divided into two groups: biochemical recurrence (BCR) and NO BCR (NBCR) group.

### 4.2. DNA Extraction

Sample resection was conducted using formalin-fixed paraffin-embedded tissues and tissue microarray from peripheral tissue of the prostate. The individual extraction of metagenomic DNA was carried out using a QIAamp DNA Mini Kit (Qiagen, Hilden, Germany) following manufacturer’s instructions. Briefly, the paraffin-embedded samples were cut and transferred into 1.5 mL tubes. For dewaxing, 1 mL xylene was added to each sample incubated at 37 C for 45 min on a shaking incubator (400 rpm). The samples were centrifuged (15,000 rpm) for 10 min and excessive xylene was removed by adding 1 mL of ethanol (70%) followed by vortexing (5 min) and centrifugation (15,000 rpm for 10 min). The remaining ethanol was removed, and samples were dried in a heat block (15 min at 37 °C). Later, 180 µL of ATL Buffer and 20 µL of Proteinase K were added to each sample followed by vigorous vortex and then incubation at 56 °C for 4 h on a shaking incubator (1000 rpm). After that 200 µL of Buffer AL was added to each tube, thoroughly vortexed and incubated at 70 °C for 10 min. Finally, the alcohol precipitation protocol was employed to purify the DNA. The quantity and quality of the extracted DNA were determined by using the Qubit-4 fluorometer (Thermo Fisher Scientific, Waltham, MA, USA) and agarose gel electrophoresis technique, respectively. Before being subjected to more investigation, the DNA samples were kept at a temperature of −20 °C. All experiments from tissue sampling (microdissection) to storage and testing were performed under sterile conditions. At this time, it was confirmed that all samples were not contaminated through PCR amplification experiments for the 16S rRNA gene at each stage in the entire process along with template-free samples.

### 4.3. Illumina Sequencing and Bioinformatics Analysis of 16S rRNA Gene Amplicons

An Illumina iSeq100 platform was used for 16S rRNA gene sequencing in keeping with a technique already described [43]. The PCR amplification of DNA was achieved using universal 16S rRNA gene primers for V4 hypervariable section following the pre-established method [44,45]. Sequences were deposited in a Sequence Read Archive (SRA) (BioProject ID: PRJNA927108, accessible at https://www.ncbi.nlm.nih.gov/sra/PRJNA927108 (accessed on 24 January 2023).

Fast length adjustment of short reads (FLASh) software (version 1.2.11) was used to combine pairs of reads from the original DNA sections [46]. Quantitative insights into microbial ecology (QIIME) were used for the sequence analysis [47]. With a resemblance of 97%, sequences were assigned to operational taxonomic units (OTUs). The appropriate illustrative sequences for each OTU were chosen, and the Ribosomal Database Project (RDP) classifier was assigned to allocate the taxonomic data [48]. Following the Human Microbiome Database, the sequences above were assigned to several phyla and species by taxonomy in variable degrees. A Bayesian approach was also used, with a 97% cutoff parameter. Bacterial heterogeneity was determined using sampling-based OTU analysis; this was shown as a rarefaction curve. The α indices, i.e., Chao 1, ACE, Simpson, Shannon, and Good’s coverage, respectively, gauged at a 3% distance, were used to assess the diversity and richness of bacteria in the samples [49,50].

A Student’s *t*-test was used to compare the bacteria’s heterogeneity within the samples. PCA using unweighted UniFrac distance measures was carried out [51]. The R package (version 4.2.2) was used to assess the interactions between the various bacterial communities within the samples. Through the use of PLS-DA, nonparametric analysis of Adonis distance matrices, and ANOSIM, the components of the bacterial populations within the specimens were evaluated [52]. Differentiating taxa amongst the two specimen cohorts at several strata was recognized using linear discriminant analysis effect size (LEfSE (version 1.1.01) (http://huttenhower.sph.harvard.edu/galaxy/ (accessed on 24 January 2023))); this software also facilitated the presentation of the data as taxonomic bar charts and cladograms [53]. The Ecological Network Analysis Pipeline was used to find network configurations within the sample bacterial populations, and Cytoscape was used to visualize the results [54]. Within the two cohorts, the functions of bacteria were forecast using the algorithm, Phylogenetic Investigation of Communities by Reconstruction of Unobserved States (PICRUSt) [55].

The MeV package was employed for data clustering and display. The operational components of the bacterial populations were predicted using the PICRUSt and the data set from the Kyoto Encyclopedia of Genes and Genomes (KEGG) [55,56]. The guidance available at https://github.com/picrust/picrust2/wiki (accessed on 9 August 2022) was used to establish the microbiome’s operational inferences using PICRUSt2 and OTUs. An analysis of variance was carried out to find any inconsistencies within the pathways [57].

## 5. Conclusions

A relative taxonomic plethora of *Lactobacillus* was seen within the NBCR specimens; these were associated with bacterial gene function alterations when contrasted against the BCR specimens. It is proposed that biochemical recurrence is related to the quantity of *Lactobacillus* present, which influences the microbiome within the prostate and synthesizes antitumor factors to confer protection against the progression of prostatic neoplasia. However, our results are from a small number of subjects, so further studies are needed. Moreover, future studies are needed to verify the role of *Lactobacillus* in the control of prostate cancer using a humanized mouse model.

## Data Availability

Data contained in the article and the original data that support the findings of the present study are available from the corresponding author upon reasonable request.

## Supplementary Materials

The supporting information can be downloaded at: https://www.mdpi.com/article/10.3390/ijms241310423/s1.

## Abbreviations

Biochemical recurrence (BCR); no biochemical recurrence (NBCR); next-generation sequencing (NGS); permutational multivariate analysis of variance (PERMANOVA); principal co-ordinate analysis (PCoA); unweighted pair group method with arithmetic mean (UPGMA); linear discriminant analysis effect size (LEfSe); phylogenetic inves-tigation of communities by reconstruction of unobserved states (PIC-RUSt); minimal set of pathways (MinPath); kyoto encyclopedia of genes and genomes (KEGG).

## References

[1] Kudo Y., Tada H., Fujiwara N., Tada Y., Tsunematsu T., Miyake Y., Ishimaru N.J.G. (2016). Environment. Oral Environ. Cancer.

[2] Perdigon G., Alvarez S., Rachid M., Agüero G., Gobbato N. (1995). Immune System Stimulation by Probiotics. J. Dairy Sci..

[3] Buermans H.P.J., den Dunnen J.T. (2014). Next generation sequencing technology: Advances and applications. BBA-Mol. Basis Dis..

[4] Ha Y.S., Kim S.Y., Chung J.I., Choi H., Kim J.H., Yu H.S., Cho I.C., Kim H.J., Chung H.C., Koh J.S. (2021). Trends in End-of-Life Resource Utilization and Costs among Prostate Cancer Patients from 2006 to 2015: A Nationwide Population-Based Study. World J. Mens Health.

[5] Braga-Basaria M., Dobs A.S., Muller D.C., Carducci M.A., John M., Egan J., Basaria S. (2006). Metabolic syndrome in men with prostate cancer undergoing long-term androgen-deprivation therapy. J. Clin. Oncol..

[6] Yang H.J., Kim J.H. (2023). Role of microbiome and its metabolite, short chain fatty acid in prostate cancer. Investig. Clin. Urol..

[7] Miyake M., Tatsumi Y., Ohnishi K., Fujii T., Nakai Y., Tanaka N., Fujimoto K. (2022). Prostate diseases and microbiome in the prostate, gut, and urine. Prostate Int..

[8] Peisch S.F., Van Blarigan E.L., Chan J.M., Stampfer M.J., Kenfield S.A. (2017). Prostate cancer progression and mortality: A review of diet and lifestyle factors. World J. Urol..

[9] Hullar M.A.J., Burnett-Hartman A.N., Lampe J.W. (2014). Gut Microbes, Diet, and Cancer. Advances in Nutrition and Cancer.

[10] Holmes E., Li J.V., Athanasiou T., Ashrafian H., Nicholson J.K. (2011). Understanding the role of gut microbiome–host metabolic signal disruption in health and disease. Trends Microbiol..

[11] Yatsunenko T., Rey F.E., Manary M.J., Trehan I., Dominguez-Bello M.G., Contreras M., Magris M., Hidalgo G., Baldassano R.N., Anokhin A.P. (2012). Human gut microbiome viewed across age and geography. Nature.

[12] Giorgetti G., Brandimarte G., Fabiocchi F., Ricci S., Flamini P., Sandri G., Trotta M.C., Elisei W., Penna A., Lecca P.G. (2015). Interactions between Innate Immunity, Microbiota, and Probiotics. J. Immunol. Res..

[13] Shinohara D.B., Vaghasia A.M., Yu S.H., Mak T.N., Bruggemann H., Nelson W.G., De Marzo A.M., Yegnasubramanian S., Sfanos K.S. (2013). A mouse model of chronic prostatic inflammation using a human prostate cancer-derived isolate of Propionibacterium acnes. Prostate.

[14] Cohen R.J., Shannon B.A., McNeal J.E., Shannon T.O.M., Garrett K.L. (2005). Propionibacterium Acnes Associated with Inflammation in Radical Prostatectomy Specimens: A Possible Link to Cancer Evolution?. J. Urol..

[15] Alfano M., Canducci F., Nebuloni M., Clementi M., Montorsi F., Salonia A. (2016). The interplay of extracellular matrix and microbiome in urothelial bladder cancer. Nat. Rev. Urol..

[16] Caini S., Gandini S., Dudas M., Bremer V., Severi E., Gherasim A. (2014). Sexually transmitted infections and prostate cancer risk: A systematic review and meta-analysis. Cancer Epidemiol..

[17] Yoon B.I., Kim S., Han D.-S., Ha U.S., Lee S.-J., Kim H.W., Han C.-H., Cho Y.-H. (2012). Acute bacterial prostatitis: How to prevent and manage chronic infection?. J. Infect. Chemother..

[18] Cox A.J., West N.P., Cripps A.W. (2015). Obesity, inflammation, and the gut microbiota. Lancet Diabetes Endocrinol..

[19] Zitvogel L., Galluzzi L., Viaud S., Vétizou M., Daillère R., Merad M., Kroemer G. (2015). Cancer and the gut microbiota: An unexpected link. Sci. Transl. Med..

[20] Mandar R. (2013). Microbiota of male genital tract: Impact on the health of man and his partner. Pharmacol. Res..

[21] Sfanos K.S., Sauvageot J., Fedor H.L., Dick J.D., De Marzo A.M., Isaacs W.B. (2008). A molecular analysis of prokaryotic and viral DNA sequences in prostate tissue from patients with prostate cancer indicates the presence of multiple and diverse microorganisms. Prostate.

[22] Khatoon J., Rai R.P., Prasad K.N. (2016). Role of Helicobacter pylori in gastric cancer: Updates. World J. Gastrointest. Oncol..

[23] Plummer M., de Martel C., Vignat J., Ferlay J., Bray F., Franceschi S. (2016). Global burden of cancers attributable to infections in 2012: A synthetic analysis. Lancet Glob. Health.

[24] Brierley J.D., Gospodarowicz M.K., Wittekind C. (2017). TNM Classification of Malignant Tumours.

[25] Cavarretta I., Ferrarese R., Cazzaniga W., Saita D., Luciano R., Ceresola E.R., Locatelli I., Visconti L., Lavorgna G., Briganti A. (2017). The Microbiome of the Prostate Tumor Microenvironment. Eur. Urol..

[26] Dallal M.M.S., Mojarrad M., Baghbani F., Raoofian R., Mardaneh J., Salehipour Z. (2015). Effects of Probiotic Lactobacillus acidophilus and Lactobacillus casei on Colorectal Tumor Cells Activity (CaCo-2). Arch. Iran. Med..

[27] Zhuo Q., Yu B.H., Zhou J., Zhang J.Y., Zhang R.L., Xie J.Y., Wang Q.L., Zhao S.L. (2019). Lysates of Lactobacillus acidophilus combined with CTLA-4-blocking antibodies enhance antitumor immunity in a mouse colon cancer model. Sci. Rep..

[28] Salva S., Marranzino G., Villena J., Aguero G., Alvarez S. (2014). Probiotic Lactobacillus strains protect against myelosuppression and immunosuppression in cyclophosphamide-treated mice. Int. Immunopharmacol..

[29] Shin R., Itoh Y., Kataoka M., Iino-Miura S., Miura R., Mizutani T., Fujisawa T. (2016). Anti-tumor activity of heat-killed Lactobacillus plantarum BF-LP284 on Meth-A tumor cells in BALB/c mice. Int. J. Food Sci. Nutr..

[30] Dai Z.W., Coker O.O., Nakatsu G., Wu W.K.K., Zhao L.Y., Chen Z.G., Chan F.K.L., Kristiansen K., Sung J.J.Y., Wong S.H. (2018). Multi-cohort analysis of colorectal cancer metagenome identified altered bacteria across populations and universal bacterial markers. Microbiome.

[31] Sugimura N., Li Q., Chu E.S.H., Lau H.C.H., Fong W., Liu W.X., Liang C., Nakatsu G., Su A.C.Y., Coker O.O. (2022). *Lactobacillus gallinarum* modulates the gut microbiota and produces anti-cancer metabolites to protect against colorectal tumourigenesis. Gut.

[32] Komiyama S., Yamada T., Takemura N., Kokudo N., Hase K., Kawamura Y.I. (2021). Profiling of tumour-associated microbiota in human hepatocellular carcinoma. Sci. Rep..

[33] Nejman D., Livyatan I., Fuks G., Gavert N., Zwang Y., Geller L.T., Rotter-Maskowitz A., Weiser R., Mallel G., Gigi E. (2020). The human tumor microbiome is composed of tumor type–specific intracellular bacteria. Science.

[34] Giralt J., Regadera J.P., Verges R., Romero J., de la Fuente I., Biete A., Villoria J., Cobo J.M., Guarner F. (2008). Effects of probiotic *Lactobacillus casei* DN-114 001 in prevention of radiation-induced diarrhea: Results from multicenter, randomized, placebo-controlled nutritional trial. Int. J. Radiat. Oncol. Biol. Phys..

[35] Singh N.K., Beckett J.M., Kalpurath K., Ishaq M., Ahmad T., Eri R.D. (2023). Synbiotics as Supplemental Therapy for the Alleviation of Chemotherapy-Associated Symptoms in Patients with Solid Tumours. Nutrients.

[36] Chandel D., Sharma M., Chawla V., Sachdeva N., Shukla G. (2019). Isolation, characterization and identification of antigenotoxic and anticancerous indigenous probiotics and their prophylactic potential in experimental colon carcinogenesis. Sci. Rep..

[37] Wang H.N., Altemus J., Niazi F., Green H., Calhoun B.C., Sturgis C., Grobmyer S.R., Eng C. (2017). Breast tissue, oral and urinary microbiomes in breast cancer. Oncotarget.

[38] Samkari A.A., Alsulami M., Bataweel L., Altaifi R., Altaifi A., Saleem A.M., Farsi A.H., Iskanderani O., Akeel N.Y., Malibary N.H. (2022). Body Microbiota and Its Relationship With Benign and Malignant Breast Tumors: A Systematic Review. Cureus J. Med. Sci..

[39] Xuan C., Shamonki J.M., Chung A., DiNome M.L., Chung M., Sieling P.A., Lee D.J. (2014). Microbial Dysbiosis Is Associated with Human Breast Cancer. PLoS ONE.

[40] Yazdi H.R., Movafagh A., Fallah F., Alizadeh Shargh S., Mansouri N., Heidary Pour A., Hashemi M. (2016). Evaluation of Methylobacterium radiotolerance and Sphyngomonas yanoikoaie in Sentinel Lymph Nodes of Breast Cancer Cases. Asian Pac. J. Cancer Prev..

[41] Kim J.H., Seo H., Kim S., Ul-Haq A., Song H.Y., Song Y.S. (2023). Malignant Prostate Tissue Is Associated with Different Microbiome Gene Functions. Diagnostics.

[42] Rickham P.P. (1964). Human experimentation. Code of ethics of the world medical association. Declaration of Helsinki. Br. Med. J..

[43] Araújo L.S.S., Silva S.Q., Teixeira M.C. (2021). Developing a biosurfactant to attenuate arsenic contamination in mining tailings. Heliyon.

[44] Ul-Haq A., Lee K.A., Seo H., Kim S., Jo S., Ko K.M., Moon S.J., Kim Y.S., Choi J.R., Song H.Y. (2022). Characteristic alterations of gut microbiota in uncontrolled gout. J. Microbiol..

[45] Ul-Haq A., Seo H., Jo S., Park H., Kim S., Lee Y., Lee S., Jeong J.H., Song H.Y. (2022). Characterization of Fecal Microbiomes of Osteoporotic Patients in Korea. Pol. J. Microbiol..

[46] Magoc T., Salzberg S.L. (2011). FLASH: Fast length adjustment of short reads to improve genome assemblies. Bioinformatics.

[47] Bolyen E., Rideout J.R., Dillon M.R., Bokulich N.A., Abnet C.C., Al-Ghalith G.A., Alexander H., Alm E.J., Arumugam M., Asnicar F. (2019). Reproducible, interactive, scalable and extensible microbiome data science using QIIME 2. Nat. Biotechnol..

[48] Bacci G., Bani A., Bazzicalupo M., Ceccherini M.T., Galardini M., Nannipieri P., Pietramellara G., Mengoni A. (2015). Evaluation of the Performances of Ribosomal Database Project (RDP) Classifier for Taxonomic Assignment of 16S rRNA Metabarcoding Sequences Generated from Illumina-Solexa NGS. J. Genom..

[49] Chao A., Lee S.M. (1992). Estimating the Number of Classes Via Sample Coverage. J. Am. Stat. Assoc..

[50] Chao A., Shen T.J. (2003). Nonparametric estimation of Shannon's index of diversity when there are unseen species in sample. Environ. Ecol. Stat..

[51] Wang L.L., Zhang F.Y., Dong W.W., Wang C.L., Liang X.Y., Suo L.L., Cheng J., Zhang M., Guo X.S., Jiang P.H. (2020). A novel approach for the forensic diagnosis of drowning by microbiological analysis with next-generation sequencing and unweighted UniFrac-based PCoA. Int. J. Leg. Med..

[52] Anderson M.J., Walsh D.C.I. (2013). PERMANOVA, ANOSIM, and the Mantel test in the face of heterogeneous dispersions: What null hypothesis are you testing?. Ecol. Monogr..

[53] Segata N., Izard J., Waldron L., Gevers D., Miropolsky L., Garrett W.S., Huttenhower C. (2011). Metagenomic biomarker discovery and explanation. Genome Biol..

[54] Deng Y., Jiang Y.H., Yang Y.F., He Z.L., Luo F., Zhou J.Z. (2012). Molecular ecological network analyses. BMC Bioinform..

[55] Douglas G.M., Beiko R.G., Langille M.G.I. (2018). Predicting the Functional Potential of the Microbiome from Marker Genes Using PICRUSt. Methods Mol. Biol..

[56] Kanehisa M., Furumichi M., Tanabe M., Sato Y., Morishima K. (2017). KEGG: New perspectives on genomes, pathways, diseases and drugs. Nucleic Acids Res..

[57] Douglas G.M., Maffei V.J., Zaneveld J.R., Yurgel S.N., Brown J.R., Taylor C.M., Huttenhower C., Langille M.G.I. (2020). PICRUSt2 for prediction of metagenome functions. Nat. Biotechnol..

