# Supplementary material for: Biochemical Recurrence in Prostate Cancer Is Associated with the Composition of Lactobacillus: Microbiome Analysis of Prostatic Tissue
Source: Int J Mol Sci. 2023 Jun 21;24(13):10423. doi: 10.3390/ijms241310423 (PMC10341780; doi:10.3390/ijms241310423)
Supplement: Supplementary file 1 [file ijms-24-10423-s001.zip › ijms-2442530-supplementary.pdf]

Supplementary data

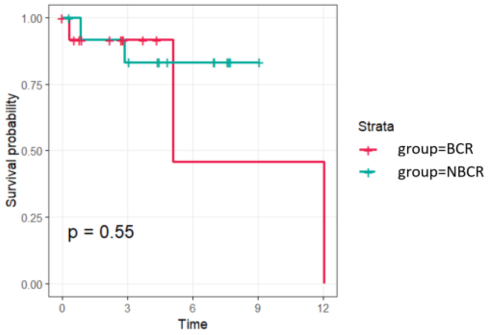

Figure S1. Kaplan–Meier survival analysis for biochemical recurrence (BCR) and with no biochemical recurrence (NBCR) .

Table S1. Clinical characteristics of patients analyzed in this study.

	NBCR (n=13)	BCR (n=13)	P value
Age	73	72	0.6084
PSA	13.5±4.5	14.9±6.6	0.8570
Gleason score			
Median	7	7	0.7213
Range	6-9	5-9	
Gleason grade			
Median	2	2	1.0000
range	1-4	1-4	
Risk stratification	Low risk = 4<br>Intermediate risk =7<br>High risk =2	Low risk=3<br>Intermediate risk=5<br>High risk =5	0.4137
Pathologic T stage	T1= 4<br>T2= 4	T1= 7<br>T2= 1	0.3591

BCR= Biochemical recurrence; NBCR= No biochemical recurrence.

Table S2. Averaged taxonomic composition for the biochemical recurrence group or no biochemical recurrence group.

Taxon rank	Taxon name	BCR	NBCR	p-value
Phylum	Proteobacteria	42.6	49.7	N.S.
	Actinobacteria	6.5	8.2	N.S.
	Bacteroidetes	26.0	19.1	N.S.
	Firmicutes	24.4	22.3	N.S.
	ETC(<1%)	0.5	0.7	
Class	Alphaproteobacteria	15.3	21.2	N.S.
	Bacteroidia	25.4	18.5	N.S.
	Bacilli	4.7	5.2	N.S.
	Actinobacteria_c	6.5	8.1	N.S.
	Gammaproteobacteria	11.3	13.5	N.S.
	Betaproteobacteria	16.0	14.9	N.S.
	Clostridia	19.4	16.9	N.S.
	ETC(<1%)	1.4	1.6	
Order	Pseudomonadales	7.4	6.6	N.S.
	Bacteroidales	25.4	18.5	N.S.
	Enterobacterales	1.2	1.5	N.S.
	Burkholderiales	15.5	14.5	N.S.
	Corynebacteriales	2.5	5.3	N.S.
	Clostridiales	19.4	16.9	N.S.
	Propionibacteriales	3.1	1.9	N.S.
	Rhizobiales	13.7	18.1	N.S.
	Bacillales	1.5	1.3	N.S.
	Xanthomonadales	2.2	5.0	N.S.
	Lactobacillales	3.2	3.9	N.S.
	Sphingomonadales	0.0	1.6	N.S.
	ETC(<1%)	4.8	5.0	
Family	Muribaculaceae	21.7	15.7	N.S.
	Staphylococcaceae	1.2	0.0	N.S.
	Comamonadaceae	14.9	12.6	N.S.
	Christensenellaceae	4.8	4.2	N.S.
	Xanthomonadaceae	2.2	5.0	N.S.
	Bacteroidaceae	1.9	1.9	N.S.
	Streptococcaceae	2.2	2.8	N.S.
	Prevotellaceae	1.6	0.0	N.S.
	Bradyrhizobiaceae	13.3	17.7	N.S.
	Propionibacteriaceae	3.1	1.9	N.S.
	Lachnospiraceae	1.8	1.6	N.S.
	Yersiniaceae	1.1	1.4	N.S.
	Corynebacteriaceae	1.6	1.6	N.S.
	Ruminococcaceae	12.7	11.0	N.S.
	Moraxellaceae	6.7	5.2	N.S.
	Lawsonella_f	0.0	2.1	N.S.
	Sphingomonadaceae	0.0	1.4	N.S.
	Mycobacteriaceae	0.0	1.6	N.S.
	Pseudomonadaceae	0.0	1.4	N.S.
	ETC(<1%)	9.0	10.9	
Genus	Cutibacterium	3.0	1.9	N.S.
	Stenotrophomonas	1.9	4.0	N.S.
	Oscillibacter	4.9	3.4	N.S.
	PAC000186_g	10.8	8.9	N.S.
	Prevotella	1.6	0.0	N.S.
	PAC001127_g	1.8	1.5	N.S.
	Serratia	1.1	1.4	N.S.
	Bacteroides	1.9	1.9	N.S.
	Christensenellaceae_uc	1.3	1.2	N.S.
	PAC001063_g	1.2	0.0	N.S.
	Streptococcus	2.2	2.7	N.S.
	Corynebacterium	1.6	1.5	N.S.
	Pseudoflavonifractor	6.6	6.3	N.S.
	PAC001360_g	1.9	1.7	N.S.
	PAC001066_g	2.9	2.4	N.S.
	Afipia	2.9	3.8	N.S.
	PAC001068_g	2.6	0.0	N.S.
	Pelomonas	14.5	11.3	N.S.
	Enhydrobacter	6.3	4.6	N.S.
	Bradyrhizobium	10.3	13.8	N.S.
	Pseudomonas	0.0	1.3	N.S.
	Lawsonella	0.0	2.1	N.S.
	Mycobacterium	0.0	1.6	N.S.
	Sphingomonas	0.0	1.4	N.S.
	JZUE_g	0.0	1.1	N.S.
	ETC(<1%)	18.7	20.2	

Relative abundances less than 1 % were expressed as ETC. Wilcoxon rank-sum test was used to analyze the significance between the two groups. N.S. Not significant.
